# Cross-cultural adaptation of Chinese international students: Effects of distant and close support-seeking

**DOI:** 10.3389/fpsyg.2023.1133487

**Published:** 2023-03-30

**Authors:** Shaofeng Zheng, Keiko Ishii

**Affiliations:** Department of Cognitive and Psychological Sciences, Graduate School of Informatics, Nagoya University, Nagoya, Japan

**Keywords:** acculturation orientation, international students, cross-cultural adaptation, social support-seeking, psychological well-being

## Abstract

**Introduction:**

Social support-seeking is considered to be an effective way for international students to cope with their acculturative stress and contribute to cross-cultural adaptation. In addition to support from people in the host country (close support), the ease of online communication now allows international students to receive additional support from people back in their home country (distant support). However, little research has investigated whether distant support works as effectively as close support. In two studies, we examined the differential effect of distant and close support-seeking on the psychological adaptation of Chinese international students in the host country and how acculturation orientations relate to the use of these two types of support.

**Methods:**

Chinese international students in Japan (Study 1; *N* = 172) and the United States (Study 2; *N* = 118) completed an online survey that assessed participants’ host/home culture orientation, distant emotional/instrumental support-seeking, close emotional/instrumental support-seeking, and psychological adaptation.

**Results:**

Results showed that distant emotional support-seeking negatively predicted psychological adaptation in the host country. Nevertheless, distant emotional support-seeking alleviated feelings of loneliness in Chinese international students as close emotional support-seeking did (Study 2). Also, the results showed that international students with higher home-culture orientation sought more distant support, whereas those with higher host-culture orientation sought more close support. Further, Chinese-culture orientation increased distant emotional support-seeking, decreasing psychological adaptation as well as loneliness.

**Discussion:**

These findings highlight the importance of considering the source and types of support when discussing the implications of social support for the cross-cultural adaptation of international students.

## 1. Introduction

International students are those who leave their own country to pursue higher education in another country. In today’s increasingly globalized society, international students represent a considerable and continuously increasingly population worldwide, particularly in developed countries (e.g., the U.S., Canada, Japan). Although the personal benefits of studying abroad are evident, including but not limited to opportunities to see the world, learn foreign languages, and expand one’s circles, the process of adapting to a new culture can be challenging and stressful. During their stay, international students have to overcome language barriers, adapt to different educational systems, establish new social circles, face discrimination, confront homesickness, and solve various practical problems (Smith and Khawaja, 2011). Social support-seeking is a powerful way for international students to ease their transition to an unfamiliar culture (Mallinckrodt and Leong, 1992). International students discuss their difficulties with their families, close friends, peers, and academic supervisors to get the emotional comfort and assistance they need, which, in turn, facilitate their adjustment in a new cultural environment.

However, recent research suggests that the effect of social support on cross-cultural adaptation is likely to depend on the geographical location of support providers (Lee et al., 2011; Demes and Geeraert, 2015). With the ease of online communication, international students can easily get additional support from people back in their country (distant support), but such support may not work as effectively as support from local people (close support). Yet little research has examined the role of distant support in psychological adaptation and its antecedent. To fill these gaps, using samples of Chinese international students in Japan and the U.S., in this paper we distinguished distant support-seeking from close support-seeking to investigate their effect on the psychological adaptation of international students, and to explore whether the frequency with which international students use each kind of support (distant or close) depends on their acculturation orientation.

### 1.1. Cross-cultural adaptation and the role of support-seeking

Cross-cultural adaptation usually is assessed by two measures: psychological and sociocultural (Ward and Kennedy, 1994; Berry, 1997). Psychological adaptation is defined as the extent to which an individual subjectively feels happy and comfortable living in the host country, whereas sociocultural adaptation refers to, at a more practical or behavioral level, how well an individual navigates daily life in the host country (Ward and Rana-Deuba, 1999). Prior research suggests that psychological adaptation and sociocultural adaptation always interrelate, though they are conceptually different and influenced by different variables (Ward and Kennedy, 1999; Demes and Geeraert, 2014). To clarify what factors contribute to international students’ adaptation to life in host cultures, several systematic reviews of papers published between 1968 and 2018 on international students’ adjustment in Western countries have been published (de Araujo, 2011; Smith and Khawaja, 2011; Zhang and Goodson, 2011b; Mesidor and Sly, 2016; Brunsting et al., 2018). Evidence from these reviews consistently suggests that social support is one of the most significant contributors to successful adaptation.

International students usually have two distinct support networks—a well-established network in their home country and a developing network in their host country. Unlike students a few decades ago, the popularization of online communication now enables international students to access their home networks as easily as their local networks. A recent meta-analysis showed that, regardless of nationality of the support providers, a greater amount of close support is associated with better psychological adaptation of international students (Bender et al., 2019). However, we know little about the implication of distant support for international students. By reminding students that they are loved and cared for by someone back in their home country, distant support may alleviate loneliness and acculturative stress of international students as effectively as close support. Overreliance on distant support, however, is hardly conducive to social connectedness with the host culture (e.g., developing new social networks), which is crucial to psychosocial adjustment (Zhang and Goodson, 2011a).

By following 2,500 international exchange students in over 50 countries, Demes and Geeraert (2015) found that close support-seeking was associated with a steady decrease of acculturative stress, whereas distant support-seeking was related to higher stress. These findings point toward the idea that distant support-seeking might be detrimental to psychological adaptation, whereas close support-seeking might promote adaptation to the host country. In line with this idea, using a sample of Chinese international students in Korea, Lee et al. (2011) found that the more Chinese students contacted people back in China, the worse they emotionally adapted to Korea. Focusing on psychological adaptation, we examined the role of distant versus close support-seeking in international students’ adaptation to the host society. We expected that distant support-seeking would reduce loneliness as much as close support-seeking though it might be negatively associated with psychological adaptation in general.

### 1.2. Acculturation orientations as predictors of support-seeking

The distinct effects of distant and close support-seeking raise another interesting question: What does influence international students’ decision about whether to seek support from close or distant networks? Social support-seeking entails self-disclosure: people must disclose their own plight voluntarily to get the support they need. Although this kind of self-disclosure carries a risk of being judged negatively (e.g., incompetent) (Lee, 2002), it also signifies trust and intimacy toward the support providers. Research on self-disclosure has suggested that people disclose more to those whom they like, and they like those who disclose themselves more (Collins and Miller, 1994). Through support transactions, people strengthen their connection with each other. Sometimes people do not ask for support just because they are not willing to develop or deepen existing relationships with specific others. Therefore, aside from the availability of support resources, whether people seek support is likely to depend on their motivation to engage with their social networks (Wright and Rains, 2013). In this case, distant versus close support-seeking is likely to be predictive of acculturation orientation.

Acculturation orientation is defined as the extent to which international students think it important or valuable to participate in the host culture (host-culture orientation), and in parallel, to maintain their heritage culture (home-culture orientation) (Berry, 1997, 2005). Depending on research purposes, the two dimensions of acculturation orientation have been studied as two independent orientations (Ryder et al., 2000; Arends-Tóth and Van de Vijver, 2007; Demes and Geeraert, 2014) or as four acculturation strategies generated from their combination (i.e., integration, assimilation, separation, and marginalization) (Berry, 2005). Due to methodology concerns (e.g., how to classify people into the four groups appropriately), the former (i.e., bidimensional approach) has been more recommended by recent work (e.g., Rudmin, 2009). Inspired by the acculturation orientation proposed by Berry (1997), many research efforts have been devoted to identifying what influences the preference for different acculturation strategies (Navas et al., 2005, 2007; Rania et al., 2019). For example, the Relative Acculturation Extended Model (RAEM) assumes that the acculturation strategies of immigrants may vary with life domains (e.g., separation in family relations but assimilation in ways of thinking) (Navas et al., 2005). In this research we adopted a bidimensional approach and investigated the independent influence of host- and home-culture orientation in support-seeking from the corresponding networks.

Acculturation orientation has important implications for the way international students balance their home networks against host networks. International students with strong home-culture orientation may value maintaining their home networks more because their home networks provide an important setting for them to engage with their heritage culture. By contrast, those with strong host-culture orientation are likely to put more effort into their host networks to integrate with the host society. In other word, home- and host-culture orientation may motivate international students to intentionally engage with the corresponding network more. Although no study has yet investigated the relation between home-culture orientation and distant social contact, the positive association between host-culture orientation and social participation in host country has been documented (Kashima and Loh, 2006; Doucerain et al., 2017; Szabó et al., 2020). For example, a longitudinal study by Doucerain et al. (2017) found that international students’ host-culture orientation, assessed on an average of 27 days after their arrival in Canada, significantly predicted more social interactions with the local group. Based on these findings, we investigated and expected that international students with strong host-culture orientation would seek more close support. By contrast, those more oriented toward their home culture might ask for more support from people in their home country to keep in touch with these well-established networks.

Beyond more social participation in the host country, research has found that host-culture orientation is positively associated with both sociocultural adaptation and psychological adaptation (Ryder et al., 2000; Cemalcilar et al., 2005; Wang and Mallinckrodt, 2006; Demes and Geeraert, 2014; Taušová et al., 2019). Regarding the association between home-culture orientation and cross-cultural adaptation, however, the findings are relatively ambiguous. Some research suggested that home-culture orientation was negatively associated with psychological adaptation (Demes and Geeraert, 2014; Taušová et al., 2019), whereas other research found that home-culture orientation was unrelated to either psychological or sociocultural adaptation (Ryder et al., 2000; Cemalcilar et al., 2005; Wang and Mallinckrodt, 2006).

Although considerable research has examined the direct effect of acculturation orientation on cross-cultural adaptation, little research has investigated the mechanisms of “how” or “why” acculturation orientation affects adaptation to the host country. The little evidence comes from a study by Zhang and Goodson (2011a). Using a sample of Chinese international students in the U.S., they found that more social interaction with Americans mediated the positive effect of host-culture orientation on psychosocial adaptation. This finding implies that acculturation orientation may influence the adaptation in host country through motivating one’s interactions with the corresponding social groups. Inspired by this, we also attempted to examine whether distant/close support-seeking would contribute to the effect of home- or host-culture orientation on psychological adaptation.

### 1.3. The current research

By conducting two surveys among Chinese international students in Japan (Study 1) and the U.S. (Study 2), we aimed to clarify the differential effect of distant and close support-seeking on psychological adaptation. Based on the distinct association of close and distant support on acculturative stress (Demes and Geeraert, 2015), we expected that close support-seeking would be positively associated with psychological adaptation (*Prediction 1*) whereas distant support-seeking would be negatively associated with psychological adaptation (*Prediction 2*). Additionally, in Study 1 for an exploratory purpose, we investigated the effect of distant and close support-seeking in sociocultural adaptation.

To understand the effect of distant support-seeking in psychological adaptation more comprehensively, in Study 2 we also assessed participants’ loneliness. Loneliness is one of the most common problems faced by international students who leave behind their well-established network to study abroad (Chataway and Berry, 1989; Sawir et al., 2008; Alharbi and Smith, 2018; Wawera and McCamley, 2020). And research has demonstrated that support-seeking effectively alleviates one’s loneliness (Zheng et al., 2021). Hence, we predicted that both distant and close support-seeking would be negatively associated with loneliness (*Prediction 3*).

Our second purpose was to investigate whether the use of distant and close support-seeking among Chinese international students would be predicted by their home- and host-culture orientation. Based on the differential significance of engaging with distant and close networks, we expected that host-culture orientation would be positively associated with close support-seeking (*Prediction 4*), whereas home-culture orientation would be positively associated with distant support-seeking (*Prediction 5*). Finally, given the relation among acculturation orientation, support-seeking, and psychological adaptation, we further investigated and predicted that more close support-seeking would mediate the positive association between host-culture orientation and psychological adaptation (*Prediction 6*), whereas more distant support-seeking would mediate the negative association between home-culture orientation and psychological adaptation (*Prediction 7*).

## 2. Study 1

### 2.1. Method

#### 2.1.1. Ethics statement

Both Studies 1 and 2 were reviewed and approved by the ethics committee at Nagoya University, Japan. All responses were kept confidential.

#### 2.1.2. Participants and procedure

We recruited 172 Chinese international students in Japan through a Chinese social network site “WECHAT.” We expected that a sample of 148 would have 80% power to detect an indirect effect based on the bias-corrected bootstrap (N > = 2,000) when the coefficients of the *a* and *b* paths are medium (Fritz and Mackinnon, 2007).

We administered an online survey to reach Chinese students living in different cities in Japan (e.g., Tokyo, Nagoya, and Osaka). The online survey was constructed in the way recommended by Regmi et al. (2016). After consenting, participants completed the measurements of stressful events, support-seeking, acculturation orientation, psychological adaptation, and sociocultural adaptation. At the end, they provided their demographic information. To promote the quality of data, we embedded two attention-check questions. Participants were allowed to withdraw from the survey at any time. However, only those who completed the full questionnaire and answered both attention-check questions correctly were included in the data analysis.

Finally, 35 participants failed the attention-check questions, and six participants did not complete the full questionnaire. Therefore, the final sample of this study included 131 participants (84 females and 47 males, *M*_age_ = 25.04, *SD* = 2.70). This sample consisted of 116 graduate students, eight undergraduates, and seven students from other educational institutions (e.g., a Japanese language school). The average length of residence in Japan was 29.19 months (*SD* = 23.27). All participants were Chinese born in China. Students took part in this survey in exchange for a $3.00 Amazon gift card.

#### 2.1.3. Measures

Using the standard forward-backward translation procedure, all measurements used in this study were translated into simplified Chinese from English by two bilingual researchers.

##### 2.1.3.1. Stressful events

To clarify the sources of stress that Chinese international students in Japan are facing, all participants first were asked to briefly describe the biggest stressor they had come across within the previous 3 months. Participants next identified the most relevant type of their own stressors from among nine options: *family relationship*, *friend relationship*, *romantic relationship*, *academic*, *health*, *financial*, *job*, *future,* or *other*.

##### 2.1.3.2. Social support-seeking

Following Demes and Geeraert (2015), using eight items revised from the two-item emotional support subscale and the two-item instrumental support subscale from Brief COPE (Carver, 1997), we assessed social support-seeking from people locally in Japan (*close support-seeking*) separately from support-seeking remotely from people back in China (*distant support-seeking*). For instance, one of the emotional support-seeking items was altered into “*I try to get emotional support from people I’ve met in Japan*” for close emotional support-seeking, and into “*I try to get emotional support from people back in China*” for distant emotional support-seeking. The sample items for instrumental support-seeking were “*I try to get help and advice from people I’ve met in Japan*” for close instrumental support-seeking, and “*I try to get help and advice from people back in China*” for distant instrumental support-seeking. Participants were asked to rate how often they tried to cope with their stressors using the strategies described by the items on a 5-point scale ranging from *not at all* (1) to *very much* (5).

Although many previous studies combined instrumental and emotional support based on their high correlation (Hendrickson et al., 2011; Lee et al., 2011; Demes and Geeraert, 2015; Hofhuis et al., 2019), to gain a more comprehensive understanding of social support-seeking we investigated emotional and instrumental support-seeking separately. Cronbach’s alpha coefficients were 0.88 for close emotional support-seeking, 0.93 for close instrumental support-seeking, 0.89 for distant emotional support-seeking, and 0.94 for distant instrumental support-seeking.

##### 2.1.3.3. Acculturation orientation

We assessed acculturation orientation using eight items revised from the Brief Acculturation Orientation Scale (BAOS) (Demes and Geeraert, 2014). Based on the existing scales (Ryder et al., 2000), Demes and Geeraert (2014) identified four main indicators of acculturation orientation: *the value of building social circles*, *taking part in traditions*, *developing cultural characteristics*, and *obeying norms.* Each indicator was presented twice (one for the home country and one for the host country). Participants were asked to rate their agreement with each statement using a 7-point scale ranging from *strongly disagree* (1) to *strongly agree* (7). Sample items include “*It is important for me to develop my Japanese characteristics*” (Japanese culture orientation) and “*It is important for me to hold on to my Chinese characteristics*” (Chinese-culture orientation). The Cronbach’s alpha coefficients were 0.80 for Chinese-culture orientation and 0.78 for Japanese-culture orientation.

##### 2.1.3.4. Psychological adaptation

We measured psychological adaptation using the Brief Psychological Adaptation Scale (BPAS) (Demes and Geeraert, 2014). The BPAS was developed to measure the stress of culture relocation (e.g., homesickness, social withdraw). It consists of 10 items covering positive and negative feelings related to cultural adaptation. We asked participants to rate the frequency of experiencing the feeling described in each item in the last 2 weeks on a 7-point scale ranging from *never* (1) to *always* (7). Sample items included “*Out of place, like you do not fit into Japanese culture*” (reversed item) and “*Happy with your day-to-day life in Japan.*” The Cronbach’s alpha coefficient was 0.80.

##### 2.1.3.5. Sociocultural adaptation

We assessed sociocultural adaptation using the Brief Sociocultural Adaptation Scale (BSAS) (Demes and Geeraert, 2014). The BSAS was developed to evaluate ease of adaptation to the social and cultural environment in the host country. Through examining the existing scales and a list of responses from a structural interview with people living abroad, Demes and Geeraert (2014) identified 12 key elements in sociocultural adaptation to the host country, such as social norms, making friends, and climate. We asked participants to rate how easy it was for them to adapt to each sociocultural element in Japan on a 7-point scale ranging from *very difficult* (1) to *very easy* (7). Sample items included “*Social norms (how to behave in public, style of clothes, what people think is funny)*” and “*Values and beliefs (what people think about religion and politics, what people think is right or wrong).*” The Cronbach’s alpha coefficient was 0.83 for our sample.

##### 2.1.3.6. Demographics

We asked participants to provide their demographic information, including (1) age, (2) gender, (3) current educational level (graduate, undergraduate, or other), (4) length of residence in Japan (months), (5) size of social network in Japan, and (6) subjective fluency of Japanese.

Size of social network in Japan was assessed by one question: “*How many friends do you have in Japan?*” And the exact number of friends participants reported were re-coded into a 7-point scale:^1^ (0) no friends; (1) equal to or more than one but less than or equal to five friends; (2) equal to or more than six but less than or equal to 10; (3) equal to or more than 11 but less than or equal to 15; (4) equal to or more than 16 but less than or equal to 20; (5) equal to or more than 21 but less than or equal to 25; (6) equal to or more than 26.

We assessed subjective fluency of Japanese using the three following questions: (1) “*What is your present level of Japanese fluency?*” (2) “*How comfortable are you communicating in Japanese?*” (3) “*How often do you communicate in Japanese?*” This method of assessing subjective language fluency has been widely used in prior research (Yeh and Inose, 2003; Sullivan and Kashubeck-West, 2015). Participants were asked to answer these three questions on a 5-point scale. The Cronbach’s alpha coefficient was 0.88.

#### 2.1.4. Data analysis

We tested predictions by multiple regression analyses with SPSS 21. Before testing the predictions, we summarized the stressors of Chinese students in Japan with a frequency analysis. We also examined Pearson correlation coefficients among the variables. Based on the patterns of correlations, we regressed distant support-seeking on Chinese-culture orientation with gender and length of residence in Japan, and we regressed close support-seeking on Japanese-culture orientation with gender, size of social network in Japan, subjective fluency of Japanese, and length of residence in Japan. Likewise, we regressed psychological/sociocultural adaptation on two culture-orientation indices and four types of support-seeking to explore how distant and close support-seeking would be associated with cross-cultural adaptation. Finally, extending the results of multiple regression analyses, we used the SPSS PROCESS macro (Model 4; Hayes, 2013) to test support-seeking as a mediator of the relation between acculturation orientation and cross-cultural adaptation. We estimated the indirect effect using a bootstrapping approach (*N* = 10,000).

### 2.2. Results

**Figure 1:**
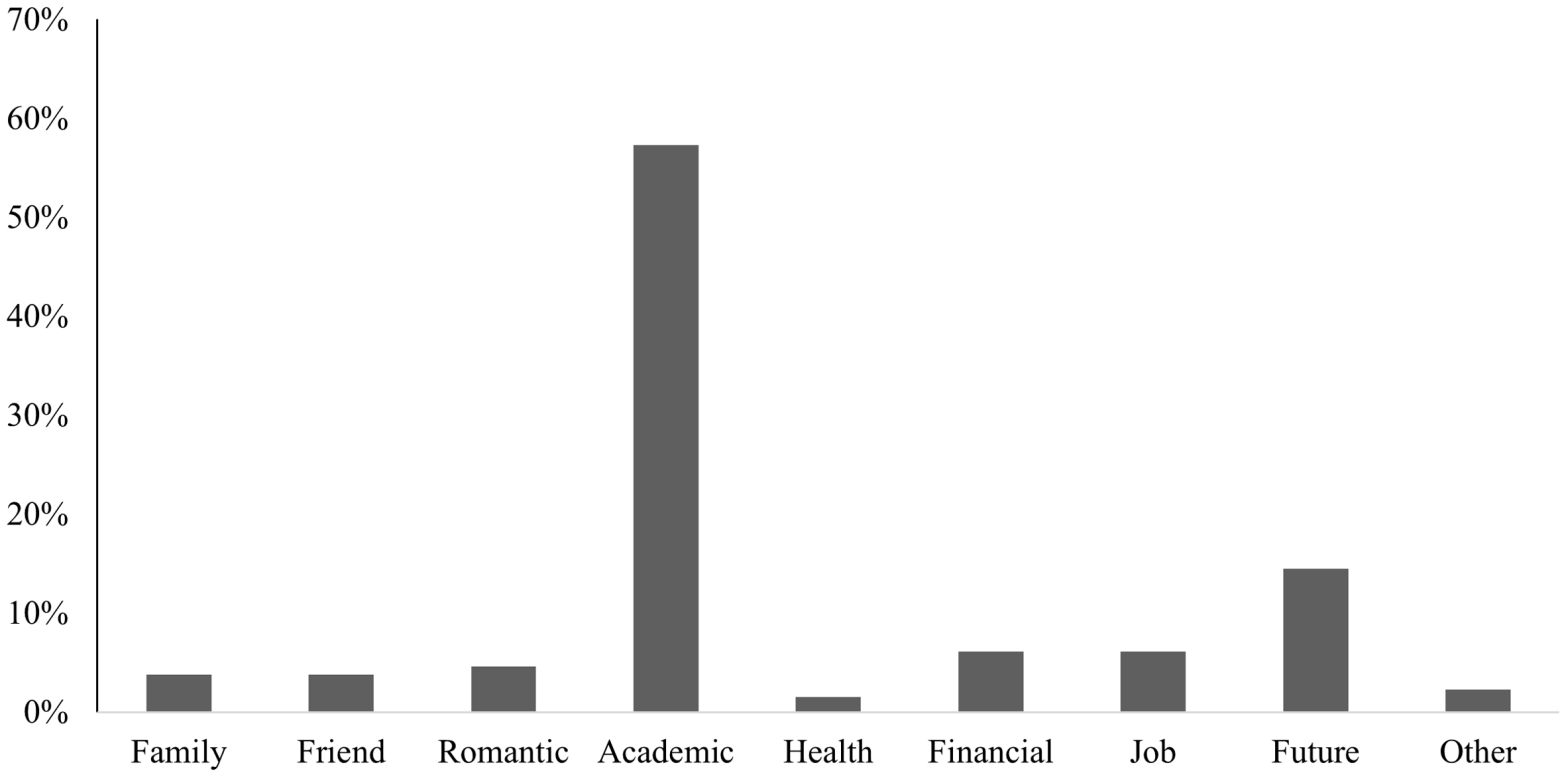
Stressors of Chinese international students in Japan.

As shown in Figure 1, the major stressors that participants faced within the previous three months were related to their academic life (57.40%) and future (14.50%). The results of the correlation analysis are presented in Table 1.

**Table 1:** Means, standard deviations, and Pearson’s correlations of all variables in Study 1.

	Mean	SD	1	2	3	4	5	6	7	8	9	10	11	12
**Demographics**														
1 Age	25.04	2.70												
2 Gender (1 = male; 0 = female)	-	-	0.11											
3 Length of residence in Japan	29.19	23.27	0.52^***^	0.04										
4 Size of network in Japan	2.43	1.50	−0.04	0.16	−0.00									
5 Subjective fluency of Japanese	3.14	1.09	0.13	0.05	0.34^***^	0.04								
**Acculturation orientation**														
6 Chinese-culture orientation	5.19	1.23	−0.14	−0.13	0.02	−0.03	0.08							
7 Japanese-culture orientation	4.40	1.16	−0.03	−0.01	0.05	0.24^**^	0.23^**^	0.10						
**Social support-seeking**														
8 Distant emotional support	3.32	1.06	−0.22^*^	−0.32^***^	−0.20^*^	−0.03	−0.14	0.29^**^	0.04					
9 Distant instrumental support	3.00	1.08	−0.22^*^	−0.25^**^	−0.17	−0.03	−0.13	0.26^**^	0.10	0.70^***^				
10 Close emotional support	2.96	1.07	0.07	−0.15	0.04	0.09	0.02	0.25^**^	0.29^**^	0.16	0.07			
11 Close instrumental support	3.09	1.03	0.00	−0.10	−0.01	0.05	0.08	0.11	0.26^**^	0.03	0.11	0.68^***^		
**Cross-cultural adaptation**														
12 Psychological adaptation	4.34	1.05	0.09	0.13	0.07	0.14	0.06	−0.22^*^	0.35^***^	−0.28^**^	−0.13	0.19^*^	0.19^*^	
13 Sociocultural adaptation	4.88	0.97	0.08	0.19^*^	0.14	0.17	0.28^**^	−0.20^*^	0.36^***^	−0.17	−0.09	0.10	0.06	0.55^***^

*N* = 131; ^*^p < 0.05, ^**^p < 0.01, ***p < 0.001.

#### 2.2.1. Social support-seeking

##### 2.2.1.1. Distant support-seeking

After controlling for the effect of gender and length of residence in Japan, the results of multiple regression analyses showed that Chinese-culture orientation significantly predicted increased distant emotional support-seeking [*b* = 0.22, SE = 0.07, *t*(127) = 3.25, *p* = 0.001] and distant instrumental support-seeking [*b* = 0.21, SE = 0.07, *t*(127) = 2.82, *p* = 0.006].

##### 2.2.1.2. Close support-seeking

Given the significant correlation of Chinese-culture orientation and close emotional support-seeking [*r*(129) = 0.25, *p* = 0.004; Table 1], we also included Chinese-culture orientation as a covariate in predicting close support-seeking besides the covariates mentioned earlier (please see the “2.1.4” for more details). The results of multiple regression analysis showed that Japanese-culture orientation significantly predicted increased close emotional support-seeking [*b* = 0.25, SE = 0.08, *t*(124) = 3.13, *p* = 0.002] and close instrumental support-seeking [*b* = 0.22, SE = 0.08, *t*(124) = 2.73, *p* = 0.007]. Unexpectedly, Chinese-culture orientation also significantly predicted seeking more emotional support from people in Japan [*b* = 0.19, SE = 0.07, *t*(124) = 2.58, *p* = 0.011].

#### 2.2.2. Cross-cultural adaptation

##### 2.2.2.1. Psychological adaptation

As shown in Table 2 (Step 2), the results of regression analyses showed that Chinese-culture orientation significantly predicted decreased psychological adaptation [*b* = −0.22, SE = 0.07, *t*(127) = −3.21, *p* = 0.002], whereas Japanese-culture orientation significantly predicted increased psychological adaptation [*b* = 0.34, SE = 0.07, *t*(127) = 4.65, *p* < 0.001]. After adding four types of social support-seeking in the regression model (Table 2, Step 3), the regression coefficients of Chinese-culture orientation [*b* = −0.20, SE = 0.07, *t*(123) = −2.88, *p* = 0.005] and Japanese-culture orientation [*b* = 0.29, SE = 0.07, *t*(123) = 3.92, *p* < 0.001] decreased. Regarding the association between social support-seeking and psychological adaptation, only two indicators of emotional support-seeking were positively associated with psychological adaptation. Specifically, more distant emotional support-seeking was significantly associated with lower psychological adaptation [*b* = −0.37, SE = 0.11, *t*(123) = −3.25, *p* = 0.002]. In contrast, more close emotional support-seeking was significantly associated with higher psychological adaptation [*b* = 0.24, SE = 0.11, *t*(132) = 2.10, *p* = 0.038].

**Table 2:** Results of hierarchical linear regression predicting psychological adaptation in Japan.

Predictors	Step 1 (*R*^2^ = 0.005, *p* = 0.431)				Step 2 (Δ*R*^2^ = 0.186, *p* < 0.001)				Step 3 (Δ*R*^2^ = 0.096, *p* = 0.004)			
	*B*	*SE*	*t(129)*	*p*	*B*	*SE*	*t(127)*	*p*	*B*	*SE*	*t(123)*	*p*
(Constant)	4.253	0.147	28.854	<0.001	3.928	0.468	8.390	<0.001	4.358	0.506	8.614	<0.001
Length of residence in Japan	0.003	0.004	0.790	0.431	0.002	0.004	0.673	0.502	0.000	0.004	−0.027	0.979
Chinese-culture orientation					−0.220	0.068	−3.208	0.002	−0.203	0.070	−2.878	0.005
Japanese-culture orientation					0.337	0.072	4.654	<0.001	0.285	0.073	3.915	<0.001
Distant emotional support									−0.369	0.114	−3.246	0.002
Distant instrumental support									0.148	0.109	1.360	0.176
Close emotional support									0.235	0.112	2.097	0.038
Close instrumental support									−0.041	0.112	−0.370	0.712

Unstandardized regression coefficients are presented.

**Figure 2:**
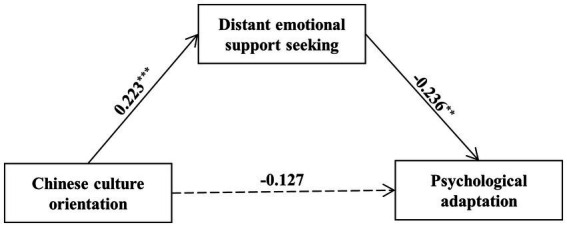
Mediation path between Chinese-culture orientation and psychological adaptation to Japan *via* distant emotional support-seeking. Gender and length of residence in Japan are included as covariates.

Furthermore, the results of mediation analyses showed that as predicted, Chinese-culture orientation fostered distant emotional support-seeking, which, in turn, reduced psychological adaptation (indirect effect = −0.05, SE = 0.03, 95% CI = [−0.12, −0.01]; Figure 2). However, the indirect effect of Japanese-culture orientation on psychological adaptation *via* close emotional support-seeking was not significant (indirect effect = 0.03, SE = 0.03, 95% CI = [−0.01, 0.11]).

##### 2.2.2.2. Sociocultural adaptation

According to the results of correlation analyses (Table 1), no indicator of social support-seeking significantly correlated to sociocultural adaptation. In this section, therefore, we examined only the predicting effect of acculturation orientation on sociocultural adaptation. The results of regression analyses showed that, after controlling for the effect of length of residence in Japan and subjective fluency in Japanese, Japanese-culture orientation positively predicted sociocultural adaptation [*b* = 0.28, SE = 0.07, *t*(126) = 4.12, *p* < 0.001], whereas Chinese-culture orientation negatively predicted sociocultural adaptation significantly [*b* = −0.20, SE = 0.06, *t*(126) = −3.25, *p* = 0.001].

### 2.3. Summary and discussion

Study 1 preliminarily demonstrated the predicted links among acculturation orientation, social support seeking, and cross-cultural adaptation by conducting a survey among Chinese students in Japan. First, Chinese-culture orientation and Japanese-culture orientation, respectively, influenced Chinese international students’ distant support-seeking and close support-seeking in Japan. When coping with daily stresses in Japan, Chinese students with significant Chinese-culture orientation exhibited a high tendency to seek support from people back in China, whereas those high in Japanese-culture orientation had a strong tendency to seek support locally from people in Japan.

Second, only emotional support-seeking had a significant effect on psychological adaptation. Specifically, consistent with our second prediction, seeking emotional support from people back in China lowered psychological adaptation, whereas seeking emotional support from people in Japan improved psychological adaptation. In addition, consistent with prior research (Demes and Geeraert, 2014), home- (i.e., Chinese) culture orientation was associated with lower psychological adaptation, whereas host- (i.e., Japanese) culture orientation was associated with higher psychological adaptation.

Third, we found evidence that the detrimental effect of Chinese-culture orientation on psychological adaptation occurred through an indirect effect on more distant emotional support-seeking. However, the indirect effect of Japanese-culture orientation on psychological adaptation *via* close support-seeking was not supported.

Finally, consistent with previous research (Demes and Geeraert, 2014), the results of Study 1 showed that host-culture orientation (i.e., Japanese-culture orientation) was associated with better sociocultural adaptation, whereas home-culture orientation (i.e., Chinese-culture orientation) was associated with worse sociocultural adaptation. However, neither distant support-seeking nor close support-seeking was correlated with sociocultural adaptation. Social support appears to be more influential in psychological adaptation than sociocultural adaptation. Although sociocultural adaptation and psychological adaptation are interrelated, their underlying mechanisms are different (Ward and Rana-Deuba, 1999). Specifically, psychological adaptation is set within a stress-coping framework which is strongly influenced by social support. By contrast, sociocultural adaptation pertains to a culture-learning framework which depends more on situational factors (e.g., cultural distance) and intercultural competence (e.g., language ability) (Ward and Rana-Deuba, 1999; Wilson et al., 2013). We also found that sociocultural adaptation (as opposed to psychological adaptation) was more related to Japanese ability [*r*(129) = 0.28, *p* < 0.001]. Given the null effect of support-seeking on sociocultural adaptation observed in Study 1, we did not further investigate it in Study 2.

These results provided initial support for the hypothesis that distant/close support-seeking of Chinese international students, to some extent, depends on their acculturation orientation. And the negative effect of home-culture orientation on psychological adaptation in the host country may be partly due to the heavy reliance on emotional support provided by people in the home country. However, the findings of Study 1 were exclusively based on Chinese international students in Japan. It has been well documented that cultural environment (i.e., collectivism vs. individualism) influences both the use and the implications of social support (Taylor et al., 2004, 2007). For example, the social norms in collectivistic culture (e.g., harmony-seeking) tend to discourage explicit support-seeking (Kim et al., 2006). And Japanese society is widely regarded as a representative of collectivism culture. Thus, we were not sure whether these findings would be applicable to Chinese international students in an individualistic society. To solve this puzzle, it would be necessary to replicate these findings with another sample in a culturally different country and obtain more evidence for our proposed explanations.

## 3. Study 2

In Study 2 we aimed to establish confidence in the main findings from Study 1 by replicating them with a sample of Chinese international students in the U.S. To unfold the effect of acculturation orientation on distant support-seeking, we also examined whether home-culture orientation would be positively associated with a motivation to maintain networks in the home country and whether this motivation would explain the association between home-culture orientation and distant support-seeking. Additionally, we assessed students’ loneliness to test *Prediction 3*–whether social support-seeking would alleviate students’ loneliness regardless of the geographical locations of support providers.

### 3.1. Method

#### 3.1.1. Participants and procedure

As in Study 1, we planned to invite about 150 Chinese international students through “WECHAT.” Finally, 118 Chinese international students in the U.S. accepted our invitations. After giving consent, participants completed a questionnaire containing three sections: the most stressful events encountered within the past 3 months, a series of self-evaluating scales (e.g., social support-seeking), and a demographics section (e.g., current academic status). We also inserted two attention-check questions.

As in Study 1, to be included in data analysis, participants were required to answer both attention-check items correctly and finish the full questionnaire. Eighteen participants did not pass the attention-check items, and two participants did not complete the full questionnaire, leaving a final total of 98 participants (64 females and 34 males, *M*_age_ = 23.89, SD = 3.09). The sample consisted of 63 graduate students, 33 undergraduate students, and two who did not indicate their current academic status. Participants reported an average length of residence in the U.S. of 51.86 months (SD = 45.01). All participants were born in China. Participants were paid $5.00 (Amazon gift card) for their participation.

#### 3.1.2. Measure

##### 3.1.2.1. Stressful events

As in Study 1, at the beginning of the survey, participants were asked to briefly describe the most stressful event they had encountered within the past three months. Subsequently, they were asked to identify which of nine stressors was more relevant to the stressful event they described: *family relationship*, *friend relationship*, *romantic relationship*, *academic*, *health*, *financial*, *job*, *future*, or *other*.

##### 3.1.2.2. Social support-seeking

We assessed **s**ocial support-seeking using eight items derived from the emotional-support subscale and the instrumental-support subscale of the Brief COPE (Carver, 1997) in the same way as Study 1 did. We assessed four types of social support-seeking: (1) close emotional support-seeking; (2) close instrumental support-seeking; (3) distant emotional support-seeking; (4) distant instrumental support-seeking. Each subscale consisted of two items. Sample items included “*I try to get help and advice from people I’ve met in the U.S.*” (close instrumental support-seeking), and “*I try to get emotional support from people I’ve met in the U.S.*” (close emotional support-seeking). Participants rated their frequency of using the strategies described in each item to cope with their daily stress on a 5-point scale (1 = *not at all*, 5 = *very much*). Cronbach’s alpha coefficients for these four subscales were good: 0.86 for close emotional support-seeking, 0.92 for close instrumental support-seeking, 0.91 for distant emotional support-seeking, and 0.87 for distant instrumental support-seeking.

##### 3.1.2.3. Acculturation orientation

We assessed acculturation orientation using the BAOS (Demes and Geeraert, 2014) as in Study 1. Sample items included: “*It is important for me to do things the way Chinese people do*” (Chinese-culture orientation), and “*It is important for me to do things the way American people do*” (American-culture orientation). Participants rated their agreement with the description of each item on a 7-point scale (1 = *strongly disagree*, 7 = *strongly agree*). The Cronbach’s alpha coefficients were 0.85 for American- culture orientation and 0.85 for Chinese-culture orientation.

##### 3.1.2.4. Maintaining network in China

We assessed maintaining network in China using the four following items: (1) “*My social network in China is very important to me,*” (2) “*It is very important for me to maintain my social circle in China,*” (3) “*My social circle is mainly in China,*” and (4) “*My circle in China is not important to me anymore*” (reversed item). Participants rated their agreement with each item on a 5-point scale (1 = *not at all*, 5 = *very much*). Cronbach’s alpha coefficient for these four items was 0.88. The items were averaged to form a composite indicator of maintaining network in China, with higher scores indicating more emphasis on maintaining circle in China.

##### 3.1.2.5. Psychological adaptation

The measure of psychological adaptation was also the same as in Study 1, using the BPAS (Demes and Geeraert, 2014). The BPAS is a 10-item scale. Participants rated how frequently they felt the way described by each item on a 7-point scale (1 = *never*, 7 = *always*). Sample items included “*Nervous about how to behave in certain situations*” (reversed item), and “*Frustrated by difficulties adapting to the U.S.*” (reversed item). Cronbach’s alpha coefficient was 0.72.

##### 3.1.2.6. Loneliness

We assessed loneliness using the Revised UCLA Loneliness Scale (R-UCLA; Russell et al., 1980). R-UCLA is a 20-item scale. Sample items included: “*People are around me but not with me,*” and “*I feel left out.*” Participants rated the frequency with which they felt the way described by the statements on a 4-point scale (1 = *never*, 4 = *often*). The Cronbach’s alpha coefficient was 0.89.

##### 3.1.2.7. Demographics

Participants self-reported their (1) current educational level, (2) length of residence in the U.S. (months), (3) size of social network in the U.S., (4) subjective fluency in English, (5) age, (6) gender, and (7) subjective socioeconomic status (SES) (Adler et al., 2000).

We assessed size of social network in the U.S. by one item: “*How many friends do you have in the U.S.?*” on a 6-point scale anchored by “*0 = no friends*” and “*6 = equal to or more than 26,*” as in Study 1.

We assessed subjective fluency in English using the same three items used in Study 1: (1) “*What is your present level of English fluency?*” (2) “*How comfortable are you communicating in English?*” (3) “*How often do you communicate in English?*” with a 5-point scale. The Cronbach’s alpha coefficient of these three items was 0.92.

#### 3.1.3. Data analysis

As in Study 1, we analyzed data using SPSS 21. First, we summarized the major stressors of Chinese students in the U.S. with a frequency analysis and tested the correlations among the variables.

Second, we used hierarchical linear-regression analyses to test the predicted effect of Chinese-culture orientation and motivation to maintain networks in China on distant support-seeking. Specifically, in Step 1 we regressed support-seeking simultaneously on Chinese-culture orientation, gender, and the length of residence in the U.S. In Step 2 we entered motivation to maintain networks in China. Then we conducted mediation analyses (PROCESS Model 4) to test the indirect effect of motivation to maintain networks in China on the relation between Chinese-culture orientation and distant support-seeking. We estimated all indirect effects in Study 2 based on 10,000 bootstrapping samples using the SPSS PROCESS macro by Hayes (2013).

Third, as in Study 1, we regressed close support-seeking on American-culture orientation with the covariates of gender, subjective fluency in English, length of residence in the U.S., and size of social network in the U.S.

Fourth, in a hierarchical linear regression analysis we tested the effect of acculturation orientation and social support-seeking on psychological adaptation. Specifically, in Step 2 we regressed psychological adaptation on Chinese-culture orientation and American-culture orientation. In Step 3 we regressed four types of social support-seeking. In Step 1 we regressed SES and length of residence in the U.S. as covariates. Then we conducted a serial mediation analysis to test whether the effect of Chinese-culture orientation on psychological adaptation would be mediated by motivation to maintain networks in China and distant emotional support-seeking in that order (PROCESS Model 6).

Likewise, in Step 1 we tested the effect of support-seeking on loneliness with size of social network in the U.S. as a covariate. Based on the results of regression analysis, we estimated the indirect effect of social support-seeking on the relation between acculturation orientation and loneliness (PROCESS Model 4).

### 3.2. Results

**Figure 3:**
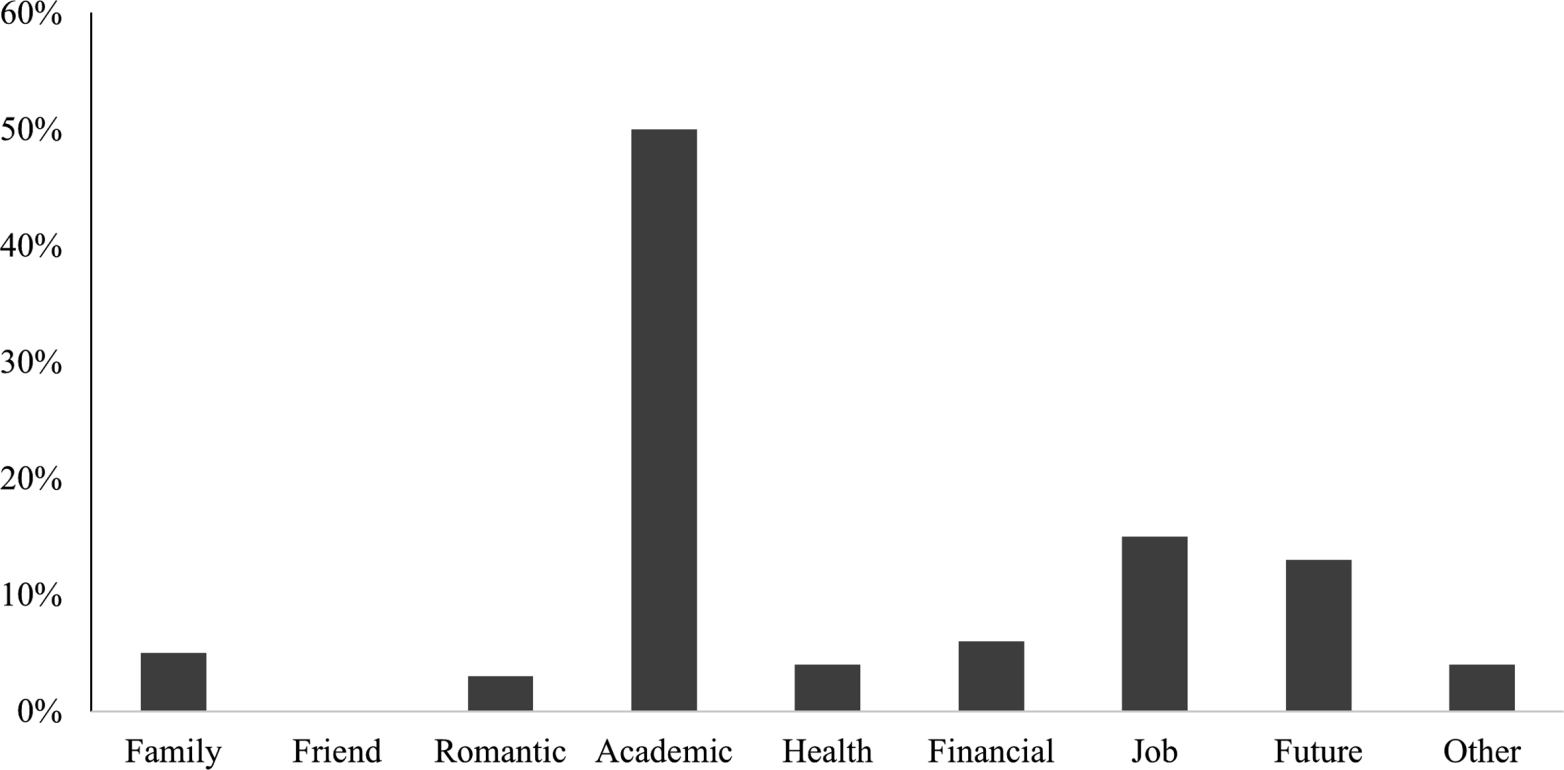
Stressors of Chinese international students in the U.S.

As shown in Figure 3, 50% of participants reported that the biggest stressors they encountered within the past 3 months originated from their academic life. And the other two common stressors were related to (part-time) jobs (15.30%) and thoughts about the future (12.20%). The results of correlation analysis are shown in Table 3.

**Table 3:** Means, standard deviations, and Pearson’s correlations of all variables in Study 2.

	Mean	*SD*	1	2	3	4	5	6	7	8	9	10	11	12	13	14
**Demographics**																
1 Age	23.89	3.09														
2 Gender (1 = male; 0 = female)	-	-	−0.16													
3 SES	5.94	1.61	−0.04	0.01												
4 Length of residence in the U.S.	51.86	45.01	0.31^**^	0.08	−0.15											
5 Size of network in the U.S.	3.24	1.60	−0.01	0.16	0.28^**^	0.07										
6 Subjective fluency of English	3.67	0.97	0.18	−0.03	0.30^**^	0.39^***^	0.40^***^									
**Acculturation orientation**																
7 Chinese-culture orientation	5.53	1.29	−0.09	0.02	−0.13	−0.21^*^	−0.06	−0.09								
8 American-culture orientation	4.56	1.29	−0.04	−0.13	0.03	0.09	−0.09	0.20^*^	0.18							
9 Maintain network in China	3.40	1.12	−0.09	0.05	0.02	−0.45^***^	0.06	−0.21^*^	0.57^***^	−0.00						
**Social support-seeking**																
10 Distant emotional support	3.05	1.14	−0.03	−0.23^*^	0.00	−0.19	−0.06	−0.08	0.40^***^	0.09	0.39^***^					
11 Distant instrumental support	2.84	1.01	−0.21^*^	−0.04	−0.01	−0.15	0.05	−0.02	0.38^***^	0.03	0.38^***^	0.76^***^				
12 Close emotional support	3.15	1.02	−0.05	−0.18	0.13	0.21^*^	0.09	0.40^***^	0.09	0.32^**^	−0.03	0.31^**^	0.26^**^			
13 Close instrumental support	3.20	1.07	−0.08	−0.07	0.11	0.16	0.16	0.31^**^	−0.04	0.21^*^	−0.03	0.28^**^	0.29^**^	0.76^***^		
**Adaptation**																
14 Psychological adaptations	4.50	0.90	−0.03	−0.02	0.30^**^	0.13	0.26^*^	0.32^**^	−0.28^**^	0.12	−0.35^***^	−0.34^**^	−0.22^*^	0.07	0.08	
15 Loneliness	2.04	0.48	−0.09	0.10	−0.36^***^	0.07	−0.33^**^	−0.39^***^	−0.27^**^	−0.16	−0.28^**^	−0.25^*^	−0.13	−0.41^***^	−0.25^*^	−0.21^*^

*N* = 98; ^*^p < 0.05, ^**^p < 0.01, ^***^p < 0.001.

#### 3.2.1. Social support-seeking

##### 3.2.1.1. Distant support-seeking

**Figure 4:**
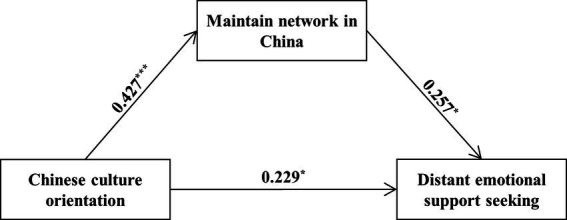
Mediation path between Chinese-culture orientation and distant emotional support-seeking *via* motivation to maintain network in China. Gender and length of residence in the U.S. are included as covariates.

As shown in Table 4, we found a positive association between Chinese-culture orientation and distant emotional support-seeking [*b* = 0.34, SE = 0.08, *t*(94) = 4.12, *p* < 0.001; Step 1]. Further, the inclusion of motivation to maintain networks in China in Step 2 significantly contributed to the explained variance in distant emotional support-seeking [Δ*R*^2^ = 0.04, *F*(1,93) = 4.52, *p* = 0.036]. As predicted, motivation to maintain networks in China significantly predicted increased distant emotional support-seeking [*b* = 0.26, SE = 0.12, *t*(93) = 2.13, *p* = 0.036]. Importantly, the result of mediation analysis showed that the positive association between Chinese-culture orientation and distant emotional support-seeking was significantly mediated by higher motivation to maintain networks in China (indirect effect = 0.11, SE = 0.06, 95% CI = [0.01, 0.24]; Figure 4).

**Table 4:** Results of hierarchical linear regression predicting distant emotional support-seeking in Study 2.

Predictors	Step 1 (*R*^2^ = 0.221, *p* < 0.001)				Step 2 (Δ*R*^2^ = 0.036, *p* = 0.036)			
	*B*	SE	*t*(94)	*p*	*B*	SE	*t*(93)	*p*
Constant	1.475	0.506	2.914	0.004	1.105	0.527	2.098	0.039
Gender	−0.547	0.217	−2.522	0.013	−0.591	0.214	−2.761	0.007
Length of residence in the U.S.	−0.002	0.002	−0.923	0.358	0.000	0.003	0.050	0.961
Chinese-culture orientation	0.339	0.082	4.123	0.000	0.229	0.096	2.391	0.019
Maintain network in China					0.257	0.121	2.126	0.036

*N* = 98; Gender: female = 0, male = 1.

We submitted distant instrumental support-seeking to the same hierarchical linear regression analysis (Table 5). As expected, Chinese culture orientation significantly predicted increased distant instrumental support-seeking [*b* = 0.29, SE = 0.08, *t*(94) = 3.76, *p* < 0.001; Step 1]. Also, motivation to maintain networks in China was significantly associated with distant instrumental support-seeking [*b* = 0.23, SE = 0.11, *t*(93) = 2.00, *p* = 0.048; Step 2]. However, the indirect effect of motivation to maintain network in China was not significant for the positive link between Chinese orientation and distant instrumental support-seeking (indirect effect = 0.10, SE = 0.05, 95% CI = [−0.01, 0.21]).

**Table 5:** Results of hierarchical linear regression predicting distant instrumental support-seeking in Study 2.

Predictors	Step 1 (*R*^2^ = 0.151, *p* = 0.001)				Step 2 (Δ*R*^2^ = 0.035, *p* = 0.048)			
	*B*	SE	*t*(94)	*p*	*B*	SE	*t*(93)	*p*
Constant	1.358	0.473	2.873	0.005	1.031	0.493	2.093	0.039
Gender	−0.093	0.202	−0.461	0.646	−0.132	0.200	−0.658	0.512
Length of residence in the U.S.	−0.002	0.002	−0.719	0.474	0.000	0.002	0.187	0.852
Chinese-culture orientation	0.288	0.077	3.758	< 0.001	0.191	0.090	2.137	0.035
Maintain network in China					0.227	0.113	2.003	0.048

*N* = 98; Gender: female = 0, male = 1.

##### 3.2.1.2. Close support-seeking

Consistent with Study 1, we found a significant predicting effect of American-culture orientation on close emotional support-seeking [*b* = 0.18, SE = 0.08, *t*(92) = 2.47, *p* = 0.015]. However, American-culture orientation was not significantly associated with close instrumental support-seeking [*b* = 0.14, SE = 0.08, *t*(92) = 1.62, *p* = 0.109].

#### 3.2.2. Cross-cultural adaptation

##### 3.2.2.1. Psychological adaptation

As predicted, we observed a significant predicting effect of Chinese-culture orientation on psychological adaptation in the U.S. [*b* = −0.17, SE = 0.07, *t*(93) = −2.48, *p* = 0.015; Step 2 in Table 6]. Unexpectedly, American-culture orientation was not significantly associated with psychological adaptation [*b* = 0.11, SE = 0.07, *t*(93) = 1.57, *p* = 0.121]. As for the predicting effect of social support-seeking, only distant emotional-support seeking was significantly associated with psychological adaptation [*b* = −0.31, SE = 0.12, *t*(89) = −2.70, *p* = 0.008]. After entering the indicators of social support-seeking in Step 3, the coefficient of Chinese-culture orientation decreased and was not significant [*b* = −0.10, SE = 0.08, *t*(89) = −1.35, *p* = 0.179].

**Table 6:** Results of hierarchical regression analysis predicting psychological adaptation in the U.S.

Predictors	Step 1 (*R*^2^ = 0.124, *p* = 0.002)				Step 2 (Δ*R*^2^ = 0.063, *p* = 0.031)				Step 3 (Δ*R*^2^ = 0.077, *p* = 0.062)			
	*B*	SE	*t*(95)	*p*	*B*	SE	*t*(93)	*p*	*B*	SE	*t*(89)	*p*
Constant	3.222	0.361	8.912	<0.001	3.925	0.598	6.567	<0.001	4.045	0.608	6.656	<0.001
SES	0.184	0.054	3.403	0.001	0.157	0.054	2.936	0.004	0.157	0.053	2.952	0.004
Length of residence in the U.S.	0.004	0.002	1.883	0.063	0.002	0.002	1.134	0.260	0.001	0.002	0.607	0.545
Chinese-culture orientation					−0.171	0.069	−2.477	0.015	−0.102	0.075	−1.354	0.179
American-culture orientation					0.105	0.067	1.565	0.121	0.100	0.068	1.470	0.145
Distant emotional support									−0.312	0.116	−2.699	0.008
Distant instrumental support									0.103	0.128	0.804	0.423
Close emotional support									0.041	0.131	0.311	0.757
Close instrumental support									0.032	0.121	0.262	0.794

*N* = 98.

**Figure 5:**
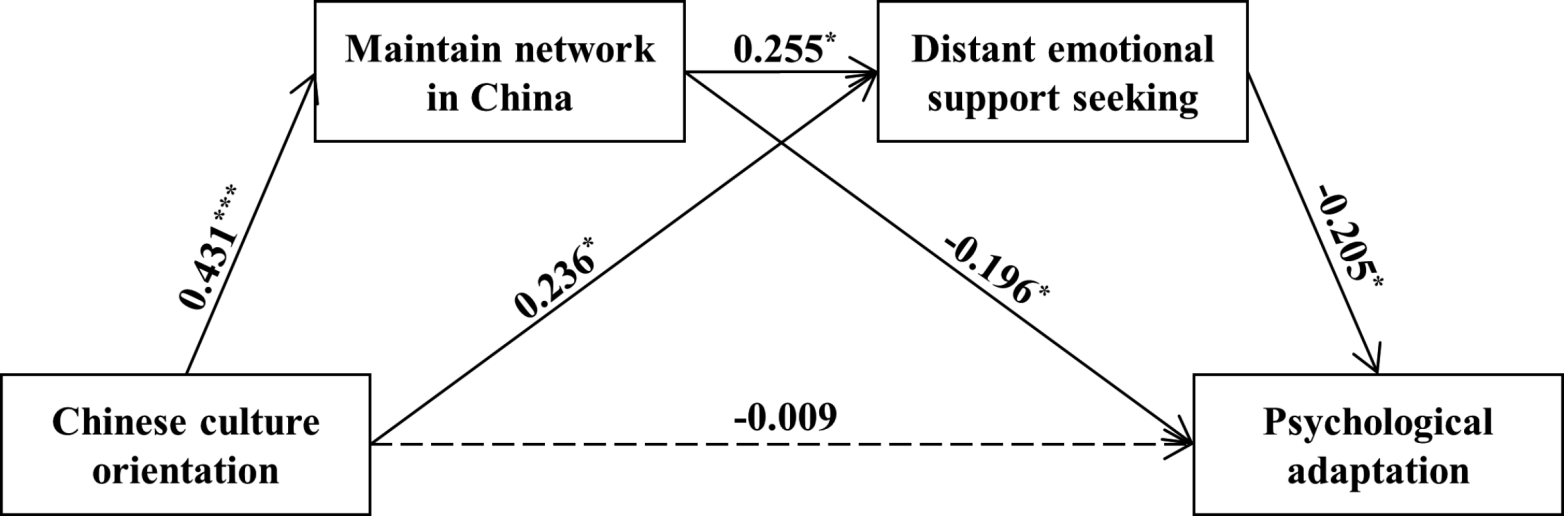
Mediation paths between Chinese-culture orientation and psychological adaptation to the U.S. *via* motivation to maintain network in China and distant emotional support-seeking. Gender, SES, and length of residence in the U.S. are included as covariates.

Furthermore, the 95% bias-corrected confidence interval based on 10,000 bootstrapping samples showed a non-zero serial indirect effect of Chinese-culture orientation on psychological adaptation in the U.S. *via* higher motivation to maintain networks in China and more distant emotional support-seeking, [−0.07, −0.01] (indirect effect = −0.02, SE = 0.02; Figure 5). And consistent with Study 1, we observed a significant indirect effect of Chinese- culture orientation on psychological adaptation through more distant emotional support-seeking (indirect effect = −0.05, SE = 0.03, 95% CI = [−0.12, −0.01]).

##### 3.2.2.2. Loneliness

The result of regression analysis showed that Chinese-culture orientation was significantly associated with loneliness [*b* = −0.10, SE = 0.04, *t*(94) = −2.79, *p* = 0.006], whereas the association between American-culture orientation and loneliness was not significant [*b* = −0.05, SE = 0.04, *t*(94) = −1.51, *p* = 0.134; Step 2 in Table 7]. Importantly, as expected, emotional support-seeking significantly predicted decreased loneliness [for distant emotional support-seeking: *b* = −0.13, SE = 0.06, *t*(90) = −2.15, *p* = 0.034; for close emotional support-seeking: *b* = −0.20, SE = 0.06, *t*(90) = −3.06, *p* = 0.003]. Distant instrumental support-seeking was positively associated with loneliness [*b* = 0.13, SE = 0.07, *t*(90) = 2.01, *p* = 0.047].

**Table 7:** Results of hierarchical regression analysis predicting loneliness in the U.S.

Predictors	Step 1 (*R*^2^ = 0.111, *p* = 0.001)				Step 2 (Δ*R*^2^ = 0.101, *p* = 0.004)				Step 3 (Δ*R*^2^ = 0.147, *p* = 0.001)			
	*B*	SE	*t*(96)	*p*	*B*	SE	*t*(94)	*p*	*B*	SE	*t*(90)	*p*
(Constant)	2.362	0.105	22.473	<0.001	3.169	0.256	12.388	<0.001	3.354	0.253	13.261	<0.001
Size of network in the U.S.	−0.101	0.029	−3.464	0.001	−0.109	0.028	−3.920	<0.001	−0.109	0.026	−4.131	<0.001
Chinese-culture orientation					−0.098	0.035	−2.785	0.006	−0.085	0.037	−2.330	0.022
American-culture orientation					−0.053	0.035	−1.513	0.134	−0.008	0.034	−0.241	0.810
Distant emotional support									−0.125	0.058	−2.153	0.034
Distant instrumental support									0.0.130	0.065	2.011	0.047
Close emotional support									−0.196	0.064	−3.062	0.003
Close instrumental support									0.055	0.061	0.897	0.372

*N* = 98.

**Figure 6:**
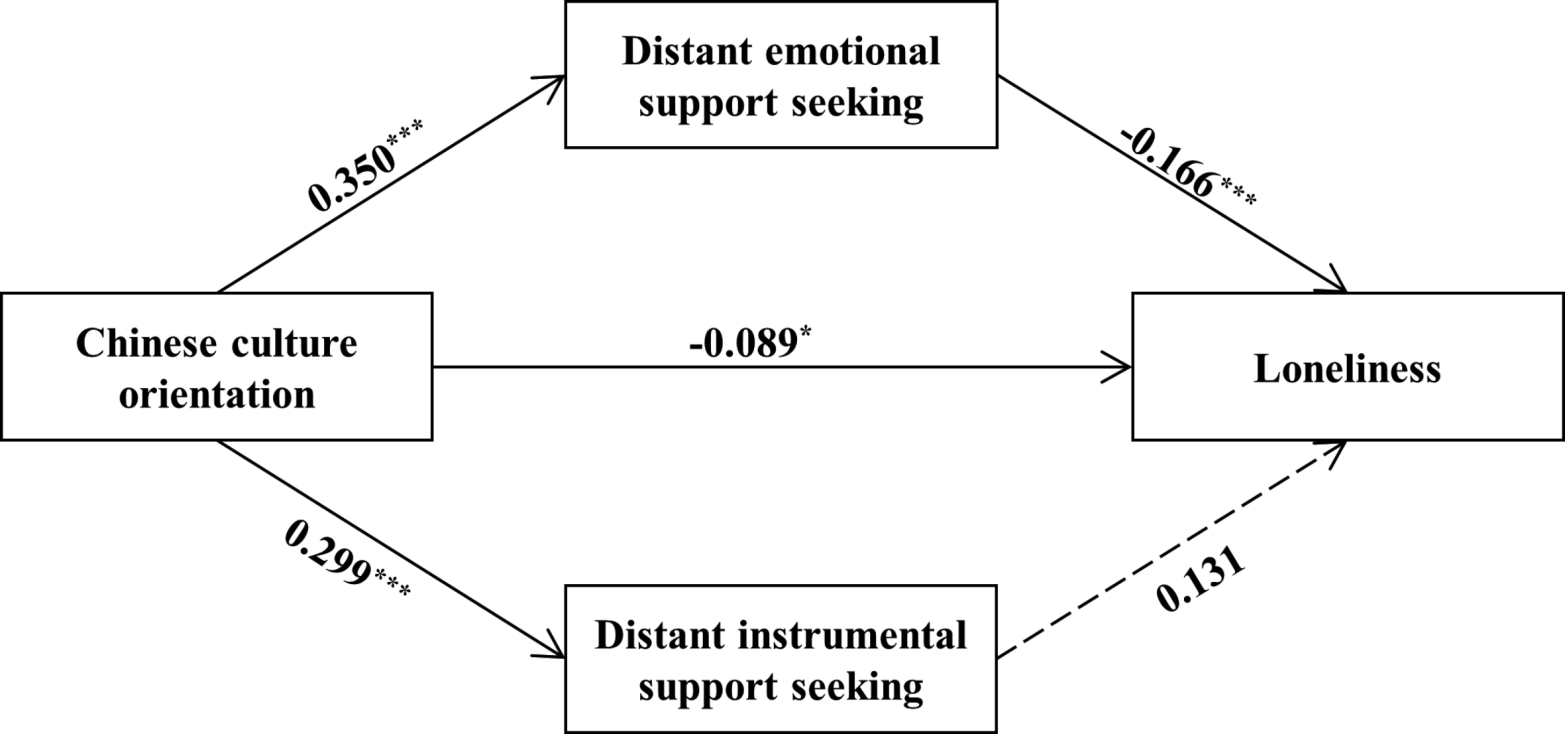
Mediation paths between Chinese-culture orientation and loneliness in the U.S. *via* distant support-seeking. Size of social network in the U.S. is included as a covariate in the model of loneliness.

To test whether the negative effect of Chinese-culture orientation on loneliness emerges *via* more distant support-seeking, we conducted a parallel mediation analysis using Model 4 of SPSS PROCESS macro, with loneliness as the outcome variable, distant emotional support-seeking and distant instrumental support-seeking as simultaneous mediators, Chinese-culture orientation as the independent variable, and size of social network in the U.S. as a covariate in the models of the outcome variable. The bootstrapping results (*N* = 10,000) indicated that more distant emotional support-seeking significantly mediated the negative association between Chinese-culture orientation and loneliness (indirect effect = −0.06, SE = 0.03, 95% CI = [−0.12, −0.01]; Figure 6).

### 3.3. Summary and discussion

The results of Study 2 confirmed the positive association between home-culture orientation and distant support-seeking among Chinese students in the U.S., showing that Chinese- culture orientation promoted emotional and instrumental support-seeking from people back in China, and that distant emotional-support-seeking mediated the association between Chinese-culture orientation and psychological adaptation. Importantly, the results of Study 2 also showed that the positive association between Chinese-culture orientation and distant emotional support was significantly mediated by students’ motivation to maintain their networks in China. In the U.S., Chinese international students with strong Chinese orientation were more likely to seek emotional support from people back in China because they had a high motivation to maintain their networks in their home country. American-culture orientation, however, only promoted emotional support-seeking from people in the U.S.

In addition to psychological adaptation, Study 2 also investigated the effect of social support-seeking on loneliness of Chinese international students in the U.S. As expected, emotional support-seeking from people in the U.S. and from people back in China both effectively alleviated Chinese international students’ loneliness. Interestingly, Chinese-culture orientation was also associated with a decrease in loneliness, which partly accounted for the increased frequency of distant emotional-support-seeking.

In sum, Study 2 replicated and extended the previous findings of the relation among home-culture orientation, distant emotional support-seeking and psychological adaptation, demonstrating that the emphasis on maintaining home culture motivates international students to maintain social networks in their home country, and this motivation prompts them to seek emotional support remotely from these networks. Distant emotional support is detrimental to psychological adaptation but helps relieve loneliness.

## 4. General discussion

By conducting two studies among Chinese international students in Japan and the U.S., we sought to understand the roles of distant and close support-seeking in psychological adaptation of international students. The results revealed that the more Chinese international students sought emotional support from people back in their home country, the less well they psychologically adapted to Japan or the U.S. Regarding the implications of close support-seeking, however, the findings of these two studies were inconsistent. In line with previous findings (Lee et al., 2011; Demes and Geeraert, 2015), among Chinese international students in Japan, seeking emotional support from people living in the host country enhanced psychological adaptation. However, this positive association was not found among Chinese international students in the U.S. Additionally, Study 2 found that both distant and close emotional support-seeking reduced international students’ loneliness. Regarding instrumental support-seeking, however, the results showed that neither close instrumental support-seeking nor distant instrumental support-seeking had significant effects on psychological adaptation.

Another major finding of our research was that Chinese international students’ distant/close support-seeking was influenced by their acculturation orientation. In line with previous studies (Zhang and Goodson, 2011a; Doucerain et al., 2017), host-culture orientation promoted support-seeking from people living in the host country, though in Study 2, American-culture orientation did not significantly predict more close instrumental support-seeking of Chinese international students in the U.S. Correspondingly, home-culture orientation (i.e., Chinese-culture orientation) promoted seeking support from people living in the home country. Finally, distant emotional support-seeking was found to mediate the effect of home-culture orientation on psychological adaptation and loneliness, whereas the association between host-culture orientation and psychological adaptation was not mediated by any type of close support-seeking.

### 4.1. Implication of distant support-seeking

Our findings were consistent with previous studies finding that international students staying in frequent contact with their home networks experienced higher acculturative stress and were emotionally worse adjusted, whereas those who interacted with people living locally exhibited better psychological adaptation in the host country (Lee et al., 2011; Demes and Geeraert, 2015).

These distinct implications of interactions with distant and close support networks are understandable. More interactions with people living locally offer international students more opportunities to learn about the host culture and increase their social connectedness with the host society, which contributes to their adaptation to a new culture. Overreliance on social networks in the home country, by contrast, may hamper the development of social networks in the host country, and the two reinforce each other in a vicious circle, which consequently increases the sense of alienation from the host society and hinders adaptation.

However, the comfort and understanding that international students receive from people back in their home country still can enhance their beliefs that they are valued and cared for by someone in the world, satisfying their emotional needs. This notion was affirmed by Study 2, in which we found that, as with close emotional support-seeking, distant emotional support-seeking helped relieve international students’ loneliness. The complicated effect of distant emotional support on psychological outcomes of cross-cultural adaptation underlines the importance of using multiple indicators to comprehensively understand the implications of support from certain networks.

### 4.2. Implication of close support-seeking

In contrast to distant support-seeking, the effect of close support-seeking in psychological adaptation seems relatively erratic. Acculturation researchers have argued that the inconsistent findings of close support might result from variations in social network diversity (Kashima and Loh, 2006; Hendrickson et al., 2011; Wang et al., 2012; Geeraert et al., 2014; Shu et al., 2020). Compared with home networks, international students’ social networks in the host country are more complex in that they consist of conationals, host nationals, and other internationals (Bochner et al., 1977). Previous studies on acculturation suggested that close support acquired from these three groups had different impacts on students’ adaptation to the host country (Kashima and Loh, 2006; Hendrickson et al., 2011; Geeraert et al., 2014; Ng et al., 2017), though the exact effect of each support group remains inconclusive. For example, research has suggested that support from the host nationals was always beneficial to international students throughout their stay, while the benefits of conational support weakened over time (Kashima and Loh, 2006; Hendrickson et al., 2011; Geeraert et al., 2014). The composition of international students’ close networks may vary with host country and from individual to individual. For example. Compared with Japan, the U.S. is relatively more multicultural and relationally mobile, accepting immigrants from all over the world including China (Kito et al., 2017). Thus, Chinese students in the U.S. may have more opportunities than those in Japan to seek support from the local Chinese community and have a more culturally diverse network. The more-diverse networks of Chinese students in the U.S. may explain the null effect of close emotional support-seeking on psychological adaptation in Study 2. To clarify further the effect of close support-seeking on cross-cultural adaptation, future research needs to differentiate the sources of close support.

### 4.3. Emotional support and instrumental support

Our research is among the first to separately investigate the implications of emotional support and instrumental support for psychological adaptation. Unexpectedly, instrumental support-seeking was not related to psychological adaptation, and in Study 2 distant instrumental-support-seeking was related to greater loneliness (Table 4). This contradicts earlier literature which suggests that, similarly to emotional support, instrumental support increases perceived support, which in turn contributes to psychological health or well-being (Taylor, 2011).

Instrumental support requires that providers offer both social and material resources. Thus, it is more costly and complicated than emotional support. First, although receiving instrumental support may effectively solve current plights, it is likely to increase pressure on support receivers to return the favor, leading to less benefit to students’ psychological well-being. For example, international students who received financial support from their families may feel more obligated to get good grades. Moreover, the backfire of instrumental support may be particularly significant among East Asians who avoid burdening others in daily life (Kim et al., 2006; Mojaverian and Kim, 2013). Previous research found that East Asians benefited more from emotional support than from instrumental support (Morling et al., 2015). Second, the function of instrumental support is likely to depend on the resources requested. For example, financial support and life suggestions may work differently in cross-cultural adaptation. Therefore, to clarify the role of instrumental support in cross-cultural adaptation, future studies might consider asking for more-directed responses such as “please describe or imagine events that make you feel financial stress.”

### 4.4. Acculturation orientation

Our findings also add more evidence to studies suggesting that acculturation orientation promotes international students’ social participations in corresponding social networks (Zhang and Goodson, 2011a; Doucerain et al., 2017; Szabó et al., 2020). Acculturation literature has suggested that a stronger orientation toward host culture is related to frequent contact or interaction with host nationals, whereas home-culture orientation is associated with frequent contact or interaction with conationals living locally (Zhang and Goodson, 2011a; Doucerain, 2018; Szabó et al., 2020).

Extending the existing studies exclusively focusing on the effect of acculturation orientation on social interaction with different national groups of close networks, our research examined the role of acculturation orientation in international students’ support-seeking toward distant and close networks. It appears that the frequency with which international students enlist support from social networks in their home or host country increased with their orientation toward the corresponding culture. This association may be explained by the motivation to develop close networks or maintain distant networks.

As developing networks in host country benefits host-culture learning, keeping in touch with networks formed in home country contributes to maintaining characteristics or identity of home culture (Hofhuis et al., 2019). Thus, the emphasis on maintaining home culture or adopting host culture is likely to increase international students’ motivation to engage with the corresponding networks. And social support-seeking is one of the effective ways to develop or maintain these networks. This speculation was supported by the findings of Study 2 that high motivation to maintain social networks in home country partly mediated the positive relationship between home-culture orientation and distant emotional support-seeking. These findings suggest that, beyond the availability of support, whether people seek support, particularly emotional support, toward specific networks depends on their social motivations, which is worth more attention in future research.

Finally, our findings also contribute to the understanding of the mechanisms underlying the association between acculturation orientation and psychological adaptation. Previous studies have indicated that host-culture orientation benefited psychological adaptation through increasing social interactions and connectedness with host national/mainstream networks (Yoon et al., 2008; Zhang and Goodson, 2011a). Extending these findings, we found that overreliance on emotional support provided by people living in the home country explained the detrimental effect of home-culture orientation on psychological adaptation. Regarding host-culture orientation, however, neither type of close support-seeking mediated its association with psychological adaptation, which might also be due to the complex composition of the close networks of international students, as discussed above. Together with the earlier findings, the present results suggest that acculturation orientation or strategies can profoundly influence international students’ attitudes and behaviors toward their social networks in home and host country, which can affect their adaption to the host culture.

### 4.5. Limitations

Despite its novel contributions, our research has several limitations. First, although we based our findings on two independent samples, the sample sizes were relatively small. Future studies with larger samples are needed to replicate these results.

Second, participants were limited to Chinese international students in Japan and the U.S. Previous research on support-seeking suggested that social support-seeking is profoundly influenced by culture (Taylor et al., 2004; Kim et al., 2006; Ishii et al., 2017; Zheng et al., 2021). East Asians are less willing than Westerners to seek social support in general (Taylor et al., 2004; Kim et al., 2006; Mojaverian et al., 2013). Thus, international students from Western countries (as opposed to East Asian countries) may be more willing and able to develop new social networks in the host country and favor close support-seeking. In addition to the willingness to seek support, the heritage culture may also influence the composition of close network in international students. We speculated that Chinese international students from a collectivist culture are likely to hang out with their compatriots more than other cultural groups and to have more conational ties in the host country. Indeed, in Study 1, we found a positive association between Chinese-cultural orientation and close emotional support-seeking. Recent research also found that the loneliness of student migrants in the Netherlands was negatively associated with the relational mobility of their heritage culture (Heu et al., 2020). Therefore, it is possible that these results are not generalizable to international students from other countries, particularly those from Western countries.

Third, our results were exclusively based on cross-sectional data, which did not permit us to draw any causal conclusions. Although the length of residence in the host country was controlled, acculturation orientation, social support-seeking, and psychological adaptation are likely to change over time and influence each other. Earlier studies have demonstrated that the initial host-culture orientation of international students could predict their social engagement during their stay (Doucerain et al., 2017). Based on these findings, although acculturation orientation is reasonably considered to be an antecedent of social interactions and adaptation, the reciprocal relationships among variables still cannot be ruled out with the cross-sectional design used. Longitudinal studies are required to confirm casual relationships further.

Finally, we use “close support” to refer to support from people living in host country. But in practice, close support is far more complicated. Unlike distant support, which is mostly provided by conational people in a remote way, close support incorporates support provided by host nationals, conationals, and other internationals either remotely or face-to-face. Prior literature in social support and acculturation has suggested that the ways in which people seek support—and which national groups they relate to—both potentially determine the effect of social support on psychological outcomes (Kashima and Loh, 2006; Huber et al., 2018). Therefore, we recommend future studies to distinguish the types of close support based on these two factors simultaneously to provide more robust and convincing evidence for the effect of close support-seeking.

## Data availability statement

The original contributions presented in the study are included in the article/Supplementary material, further inquiries can be directed to the corresponding author.

## Ethics statement

The studies involving human participants were reviewed and approved by Nagoya University. The patients/participants provided their written informed consent to participate in this study.

## Author contributions

SZ and KI conceptualized and designed the research together. SZ collected data and wrote the first draft. KI provided insightful comments and edited the manuscript. All authors contributed to the article and approved the submitted version.

## Funding

This research was supported by the Grant-in-Aid for JSPS (Japan Society for the Promotion of Science) Research Fellows (21 J13794).

## Conflict of interest

The authors declare that the research was conducted in the absence of any commercial or financial relationships that could be construed as a potential conflict of interest.

## Publisher’s note

All claims expressed in this article are solely those of the authors and do not necessarily represent those of their affiliated organizations, or those of the publisher, the editors and the reviewers. Any product that may be evaluated in this article, or claim that may be made by its manufacturer, is not guaranteed or endorsed by the publisher.

## Footnotes

^1^Even if the exact number of friends is used, the significant results of the following regression analyses remain.
